# Association of pre-treatment lymphocyte-monocyte ratio with survival outcome in patients with head and neck cancer treated with chemoradiation

**DOI:** 10.1186/s12885-023-11062-3

**Published:** 2023-06-21

**Authors:** Brian Yu, Sung Jun Ma, Michael Khan, Jasmin Gill, Austin Iovoli, Fatemeh Fekrmandi, Mark K. Farrugia, Kimberly Wooten, Vishal Gupta, Ryan McSpadden, Moni A. Kuriakose, Michael R. Markiewicz, Ayham Al-Afif, Wesley L. Hicks, Mukund Seshadri, Andrew D. Ray, Elizabeth A. Repasky, Anurag K. Singh

**Affiliations:** 1 Jacobs School of Medicine and Biomedical Sciences, University at Buffalo, The State University of New York, 955 Main Street, Buffalo, NY 14203 USA; 2 Department of Radiation Medicine, Roswell Park Comprehensive Cancer Center, 665 Elm Street, Buffalo, NY 14203 USA; 3 University at Buffalo, The State University of New York, 12 Capen Hall, Buffalo, NY 14260 USA; 4 Department of Head and Neck Surgery, Roswell Park Comprehensive Cancer Center, 665 Elm Street, Buffalo, NY 14203 USA; 5 Department of Oral and Maxillofacial Surgery, School of Dental Medicine, University at Buffalo, The State University of New York, 3435 Main Street, Buffalo, NY 14214 USA; 6 Department of Neurosurgery, Jacobs School of Medicine and Biomedical Sciences, University at Buffalo, The State University of New York, 955 Main Street, Buffalo, NY 14203 USA; 7 Department of Oral Oncology, Roswell Park Comprehensive Cancer Center, 665 Elm Street, Buffalo, NY 14203 USA; 8 Department of Cancer Prevention and Control, Roswell Park Comprehensive Cancer Center, 665 Elm Street, Buffalo, NY 14203 USA; 9 Department of Immunology, Roswell Park Comprehensive Cancer Center, 665 Elm Street, Buffalo, NY 14203 USA

**Keywords:** LMR, Lymphocyte, Monocyte, chemoRT, HN cancer, HPV

## Abstract

**Background:**

Given the role of systematic inflammation in cancer progression, lymphocyte-monocyte ratio (LMR) from peripheral blood has been suggested as a biomarker to assess the extent of inflammation in several solid malignancies. However, the role of LMR as a prognostic factor in head and neck cancer was unclear in several meta-analyses, and there is a paucity of literature including patients in North America. We performed an observational cohort study to evaluate the association of LMR with survival outcomes in North American patients with head and neck cancer.

**Methods:**

A single-institution, retrospective database was queried for patients with non-metastatic head and neck cancer who underwent definitive chemoradiation from June 2007 to April 2021 at the Roswell Park Comprehensive Cancer Center. Primary endpoints were overall survival (OS) and cancer-specific survival (CSS). The association of LMR with OS and CSS was examined using nonlinear Cox proportional hazard model using restricted cubic splines (RCS). Cox multivariable analysis (MVA) and Kaplan–Meier method were used to analyze OS and CSS. Pre-radiation LMR was then stratified into high and low based on its median value. Propensity scored matching was used to reduce the selection bias.

**Results:**

A total of 476 patients met our criteria. Median follow up was 45.3 months (interquartile range 22.8–74.0). The nonlinear Cox regression model showed that low LMR was associated with worse OS and CSS in a continuous fashion without plateau for both OS and CSS. On Cox MVA, higher LMR as a continuous variable was associated with improved OS (adjusted hazard ratio [aHR] 0,90, 95% confidence interval [CI] 0.82–0.99, *p* = 0.03) and CSS (aHR 0.83, 95% CI 0.72–0.95, *p* = 0.009). The median value of LMR was 3.8. After propensity score matching, a total of 186 pairs were matched. Lower LMR than 3.8 remained to be associated with worse OS (HR 1.59, 95% CI 1.12–2.26, *p* = 0.009) and CSS (HR 1.68, 95% CI 1.08–2.63, *p* = 0.02).

**Conclusion:**

Low LMR, both as a continuous variable and dichotomized variable, was associated with worse OS and CSS. Further studies would be warranted to evaluate the role of such prognostic marker to tailor interventions.

## Introduction

Inflammationplays a critical role in both the progression of cancer and its response to therapies [1, 2]. There has been a recent focus on exploring inflammatory markers as a prognostic factor for cancer-related outcomes as they are inexpensive, non-invasive, and minimize complications for the patient [3]. These markers are of particular interest in human papillomavirus (HPV)-negative head and neck cancers, where no widely accepted prognostic biomarkers exist [3]. One such marker is lymphocyte-monocyte ratio (LMR). The use of LMR as a prognostic factor in head and neck cancer is equivocal in a recent meta-analysis displaying conflicting findings [4].

To date, there have been no studies evaluating the utility of LMR as a prognostic factor for head and neck cancer within North America. The majority of studies were performed in China, Japan, and the United Kingdom, with inconsistent use of smoking history as a pertinent risk factor in a recent meta-analysis [4]. Current studies may not be fully applicable to North America due to differential HPV distribution and differences in prevalence of other risk factors such as smoking and alcohol use [5–7]. Recent in-vitro studies have found p16-mediated inflammatory microenvironments in models of HPV positive cancer which may contribute to differential inflammatory profiles between HPV positive and HPV negative cohorts [8]. In addition, there has been no subset analysis of HPV positive and HPV negative head and neck cancers, which vary greatly in their outcomes [9]. To address this knowledge gap, we performed an observational cohort study to evaluate the association of LMR and survival outcomes in North American patients with head and neck cancer.

## Materials and methods

Roswell Park Comprehensive Cancer Center institutional review board approved our study (EDR 103707). Our study complies with the Strengthening the Reporting of Observational Studies in Epidemiology (STROBE) reporting guideline.

A single-institution, retrospective database was queried for patients with non-metastatic head and neck cancer who underwent curative-intent definitive chemoradiation from June 2007 to April 2021 at the Roswell Park Comprehensive Cancer Center. Intensity modulated radiation therapy (IMRT) with 70 Gy to gross disease and 56 Gy to elective neck lymph nodes in 35 fractions [10]. Patients were excluded if they underwent radiation alone, induction chemotherapy, postoperative radiation, or did not have LMR or survival data.

Variables of interest used in this study included pre-treatment LMR, age, race, gender, smoking status, Karnofsky Performance Status (KPS), number of comorbidities, primary disease site, tumor T and N staging based on the American Joint Committee on Cancer (AJCC) 7^th^ edition, HPV status based on p16 status, and chemotherapy agent. Comorbidities included respiratory (e.g., chronic obstructive pulmonary disease), genitourinary (e.g., chronic kidney disease), endocrine (e.g., diabetes, hypothyroidism), cardiovascular (e.g., hypertension, stroke), and gastrointestinal systems (e.g., gastroesophageal reflux disease). For analysis, missing values were coded as unknown. Races are self-identified as African American, American Indian/Alaska Native, Asian, Hispanic, unknown or declined to answer, and White. Given the small subgroup sample sizes, non-White patients were grouped together as a single category.

Primary endpoints were overall survival (OS) and cancer-specific survival (CSS), defined as time intervals from diagnosis to death from any cause or cancer-related death respectively. Other endpoints included progression-free survival (PFS), locoregional failure (LRF), and distant failure (DF). PFS was defined as time interval from diagnosis to either death from any cause or tumor progression. LRF and DF were defined as time intervals from diagnosis to tumor recurrences in head and neck or outside the head and neck, respectively. All tumor recurrences were confirmed based on multidisciplinary discussion using radiographic findings and, if applicable, biopsy results of metastatic sites. For those with multiple failure events either synchronously or metachronously during their follow up period, all failure events were counted separately for analysis.

### Statistical analysis

Peripheral complete blood count data was used to calculate pre-treatment LMR. The association of LMR with OS and CSS was examined using nonlinear Cox proportional hazard model using restricted cubic splines (RCS) with 3 knots at the 10^th^, 50^th^, and 90^th^ percentiles based on the lowest Akaike information criterion [11, 12] as previously shown [13].

Cox multivariable analysis (MVA) and Kaplan–Meier method were used to analyze OS, CSS, and PFS using LMR as a continuous variable. Pre-radiation LMR was then stratified into high and low based on its median value. Logistic MVA was performed to identify variables associated with low LMR below its median value. Fine-Gray MVA was performed to analyze LRF and DF outcomes with death as a competing event. MVA models included all of the variables listed previously. Among those with available HPV data for oropharyngeal cancer, subgroup analysis was performed. In addition, given the prognostic role of neutrophil counts from peripheral blood on treatment outcomes [14, 15], another subgroup analysis including absolute neutrophil count (ANC) was performed. Propensity scored matching between high versus low LMR based on its median value was performed to construct matched pairs based on nearest neighbor method in a 1:1 ratio with no replacement using a caliper distance of 0.2 [16]. Standardized means differences for all matched variables were less than 0.1, suggesting negligible differences [17]. Matched variables included all variables previously included for MVA. Cox regression model was used to evaluate OS and CSS after matching.

P values less than or equal to 0.05 were considered statistically significant. All p values were two-sided. Analyses was performed using R (version 4.1.2, R Project for Statistical Computing, Vienna, Austria).

## Results

A total of 476 patients (391 male [82.1%], median [interquartile range] age, 61 [55–67] years) met our criteria (Table 1). Median follow up was 45.3 months (interquartile range 22.8–74.0). Most patients were White (*n* = 414, 87.0%) with favorable performance status (KPS 90–100: *n* = 348, 73.1%) and had HPV-associated squamous cell carcinoma (*n* = 231, 48.5%) in oropharynx (*n* = 272, 57.1%) treated with cisplatin as concurrent chemotherapy regimen (*n* = 403, 84.7%).

**Table 1:** Baseline characteristics

	Before matching					After matching				
	3.8 or higher		< 3.8			3.8 or higher		< 3.8		
	*N*	%	*N*	%	*P*	*N*	%	*N*	%	*P*
Gender					0.07					0.89
Male	183	78.9	208	85.2		155	83.3	153	82.3	
Female	49	21.1	36	14.8		31	16.7	33	17.7	
Smoker					0.30					0.49
Never/Former	192	82.8	192	78.7		151	81.2	157	84.4	
Current	40	17.2	52	21.3		35	18.8	29	15.6	
Age					0.48					1
< 65	166	71.6	167	68.4		131	70.4	131	70.4	
65 or older	66	28.4	77	31.6		55	29.6	55	29.6	
KPS					0.29					1
< 90	53	22.8	71	29.1		46	24.7	46	24.7	
90–100	177	76.3	171	70.1		138	74.2	138	74.2	
Not available	2	0.9	2	0.8		2	1.1	2	1.1	
Race					0.02					1
White	193	83.2	221	90.6		165	88.7	166	89.2	
Other	39	16.8	23	9.4		21	11.3	20	10.8	
Comorbidity					0.90					1
0	35	15.1	40	16.4		30	16.1	30	16.1	
1–3	140	60.3	147	60.2		113	60.8	114	61.3	
> 3	57	24.6	57	23.4		43	23.1	42	22.6	
Site					0.50					0.75
Oropharynx	131	56.5	141	57.8		103	55.4	108	58.1	
Larynx	60	25.9	53	21.7		44	23.7	45	24.2	
Other	41	17.7	50	20.5		39	21.0	33	17.7	
T staging					0.008					0.76
1–2	133	57.3	110	45.1		97	52.2	93	50.0	
3–4	99	42.7	134	54.9		89	47.8	93	50.0	
N staging					0.84					0.65
0–1	70	30.2	71	29.1		55	29.6	50	26.9	
2–3	162	69.8	173	70.9		131	70.4	136	73.1	
HPV					0.07					0.75
Negative	35	15.1	53	21.7		33	17.7	28	15.1	
Positive	124	53.4	107	43.9		93	50.0	94	50.5	
Not available	73	31.5	84	34.4		60	32.3	64	34.4	
Chemo					0.25					0.77
Cisplatin	201	86.6	202	82.8		160	86.0	157	84.4	
Other	31	13.4	42	17.2		26	14.0	29	15.6	

*KPS* Karnofsky performance status, *HPV* human papillomavirus

**Fig. 1:**
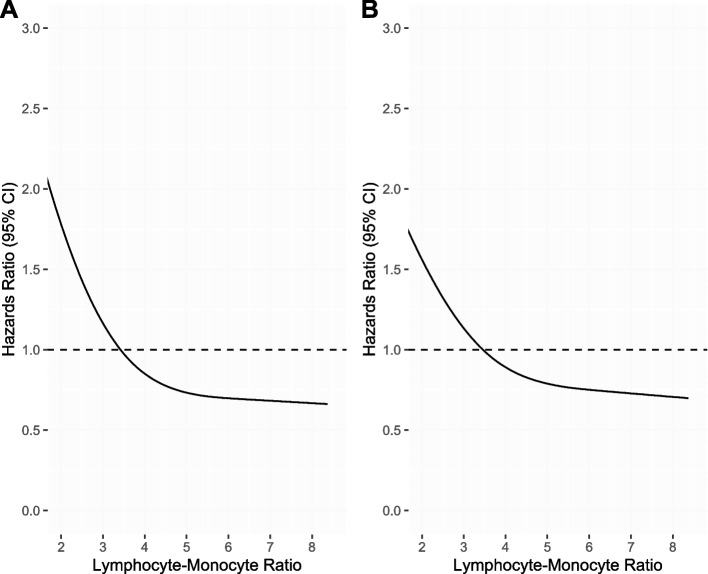
Nonlinear Cox regression model using restricted cubic spline for the association between lymphocyte-monocyte ratio and survival outcomes

The nonlinear Cox regression model using RCS method showed that low LMR was associated with worse OS and CSS in a continuous fashion without plateau and crossed the hazard ratio of 1 at LMR 3.4 for both OS and CSS outcomes (Fig. 1). On Cox MVA, higher LMR was associated with improved OS (adjusted hazard ratio [aHR] 0.90, 95% confidence interval [CI] 0.82–0.99, *p* = 0.03) and CSS (aHR 0.83, 95% CI 0.72–0.95, *p* = 0.009; Table 2). However, it was not associated with PFS (aHR 0.93, 95% CI 0.86–1.01, *p* = 0.09), LRF (aHR 0.89, 95% CI 0.75–1.05, *p* = 0.18), or DF (aHR 0.94, 95% CI 0.81–1.08, *p* = 0.39; Table 3).

**Table 2:** Cox multivariable analysis for overall survival and cancer-specific survival

	Overall survival			Cancer-specific survival		
	aHR	95% CI	*P*	aHR	95% CI	*P*
LMR						
For every increase by 1	0.90	0.82–0.99	0.03	0.83	0.72–0.95	0.009
Gender						
Male	Reference			Reference		
Female	1.05	0.68–1.61	0.83	0.89	0.51–1.57	0.7
Smoker						
Never/Former	Reference			Reference		
Current	1.7	1.15–2.49	0.007	1.4	0.86–2.28	0.17
Age						
For every increase by 1	1.03	1.01–1.05	0.002	1.03	1.00–1.05	0.02
KPS						
< 90	Reference			Reference		
90–100	0.7	0.49–1.01	0.05	0.46	0.29–0.71	< 0.001
Not available	< 0.001	0.00-Infinity	0.99	< 0.001	0.00-Infinity	1
Race						
White	Reference			Reference		
Other	1.6	1.04–2.48	0.03	1.73	1.01–2.97	0.05
Comorbidity						
0	Reference			Reference		
1	0.54	0.32–0.91	0.02	0.52	0.28–0.99	0.05
2	0.88	0.51–1.53	0.65	0.6	0.30–1.21	0.15
3	0.27	0.15–0.51	< 0.001	0.26	0.12–0.55	< 0.001
> 3	0.85	0.50–1.44	0.54	0.68	0.35–1.31	0.25
Site						
Oropharynx	Reference			Reference		
Larynx	1	0.62–1.61	1	1.13	0.62–2.08	0.69
Other	1.07	0.67–1.69	0.78	1.38	0.77–2.48	0.28
T staging						
1–2	Reference			Reference		
3–4	2.19	1.54–3.11	< 0.001	3.45	2.14–5.54	< 0.001
N staging						
0–1	Reference			Reference		
2–3	1.8	1.19–2.74	0.006	2.59	1.50–4.49	< 0.001
HPV						
Negative	Reference			Reference		
Positive	0.65	0.40–1.07	0.09	0.74	0.39–1.39	0.35
Not available	1.04	0.70–1.57	0.83	1.04	0.62–1.74	0.88
Chemo						
Cisplatin	Reference			Reference		
Other	1.43	0.90–2.28	0.13	1.41	0.77–2.59	0.27

*LMR* lymphocyte-monocyte ratio, *aHR* adjusted hazards ratio, *95% CI* 95% confidence interval, *KPS* Karnofsky performance status, *HPV* human papillomavirus

**Table 3:** Cox multivariable analysis for progression-free survival and Fine-Gray multivariable analysis for locoregional and distant failures

	Progression-Free Survival			Locoregional Failure			Distant Failure		
	aHR	95% CI	*P*	aHR	95% CI	*P*	aHR	95% CI	*P*
LMR									
For every increase by 1	0.93	0.86–1.01	0.09	0.89	0.75–1.05	0.18	0.94	0.81–1.08	0.39
Gender									
Male	Reference			Reference			Reference		
Female	1.03	0.70–1.52	0.89	1.06	0.53–2.09	0.87	0.46	0.20–1.03	0.06
Smoker									
Never/Former	Reference			Reference			Reference		
Current	1.51	1.06–2.15	0.02	1.08	0.57–2.05	0.81	1.4	0.78–2.53	0.26
Age									
For every increase by 1	1.02	1.00–1.04	0.02	1.01	0.98–1.04	0.54	1	0.98–1.03	0.92
KPS									
< 90	Reference			Reference			Reference		
90–100	0.81	0.58–1.14	0.22	0.91	0.49–1.68	0.76	0.59	0.33–1.03	0.06
Not available	< 0.001	0.00-Infinity	0.99	< 0.001	0.00-Infinity	1	< 0.001	0.00-Infinity	1
Race									
White	Reference			Reference			Reference		
Other	1.4	0.93–2.11	0.11	2.23	1.16–4.29	0.02	1.45	0.74–2.86	0.28
Comorbidity									
0	Reference			Reference			Reference		
1	0.57	0.35–0.92	0.02	0.55	0.24–1.30	0.17	0.96	0.43–2.13	0.92
2	0.81	0.49–1.34	0.41	0.87	0.35–2.18	0.77	1.12	0.48–2.64	0.79
3	0.33	0.19–0.57	< 0.001	0.58	0.24–1.40	0.22	0.39	0.15–1.02	0.06
> 3	0.74	0.46–1.20	0.23	0.42	0.16–1.07	0.07	1.1	0.48–2.52	0.82
Site									
Oropharynx	Reference			Reference			Reference		
Larynx	1.06	0.68–1.63	0.81	1.29	0.58–2.86	0.53	1.52	0.73–3.17	0.27
Other	1.11	0.73–1.69	0.62	1.45	0.66–3.18	0.36	1.79	0.93–3.45	0.08
T staging									
1–2	Reference			Reference			Reference		
3–4	1.97	1.44–2.70	< 0.001	2.6	1.35–5.02	0.004	2.75	1.62–4.66	< 0.001
N staging									
0–1	Reference			Reference			Reference		
2–3	1.94	1.31–2.86	< 0.001	1.21	0.62–2.35	0.58	4.64	2.21–9.73	< 0.001
HPV									
Negative	Reference			Reference			Reference		
Positive	0.56	0.36–0.88	0.01	0.4	0.16–0.98	0.05	1.11	0.52–2.35	0.8
Not available	0.86	0.59–1.26	0.44	0.9	0.47–1.72	0.76	0.93	0.47–1.87	0.84
Chemo									
Cisplatin	Reference			Reference			Reference		
Other	1.61	1.05–2.47	0.03	1.09	0.44–2.67	0.86	2.36	1.23–4.52	0.01

*LMR* lymphocyte-monocyte ratio, *aHR* adjusted hazards ratio, *95% CI* 95% confidence interval, *KPS* Karnofsky performance status, *HPV* human papillomavirus

The median value of LMR was 3.8. On logistic MVA (Table 4), patients with other racial background (adjusted odds ratio [aOR] 0.85, 95% CI 0.74–0.97, *p* = 0.02) and positive HPV status (aOR 0.82, 95% CI 0.72–0.94, *p* = 0.005) were less likely to have low LMR. Higher T staging was associated with low LMR (aOR 1.15, 95% CI 1.04–1.27, *p* = 0.005).

**Table 4:** Logistic multivariable analysis for Lymphocyte-Monocyte Ratio

	aOR	95% CI	*P*
Gender			
Male	Reference		
Female	0.88	0.78–1.00	0.05
Smoker			
Never/Former	Reference		
Current	1.04	0.92–1.16	0.56
Age			
< 65	Reference		
65 or older	1.03	0.93–1.14	0.61
KPS			
< 90	Reference		
90–100	0.94	0.84–1.04	0.23
Not available	0.97	0.59–1.59	0.9
Race			
White	Reference		
Other	0.85	0.74–0.97	0.02
Comorbidity			
0	Reference		
1–3	0.93	0.82–1.06	0.3
> 3	0.91	0.78–1.06	0.21
Site			
Oropharynx	Reference		
Larynx	0.87	0.75–1.00	0.05
Other	1	0.88–1.14	1
T staging			
1–2	Reference		
3–4	1.15	1.04–1.27	0.005
N staging			
0–1	Reference		
2–3	1.04	0.93–1.17	0.47
HPV			
Negative	Reference		
Positive	0.82	0.72–0.94	0.005
Not available	0.93	0.82–1.06	0.26
Chemo			
Cisplatin	Reference		
Other	1.12	0.98–1.27	0.1

*aOR* adjusted odds ratio, *95% CI* 95% confidence interval, *KPS* Karnofsky performance status, *HPV* human papillomavirus

**Fig. 2:**
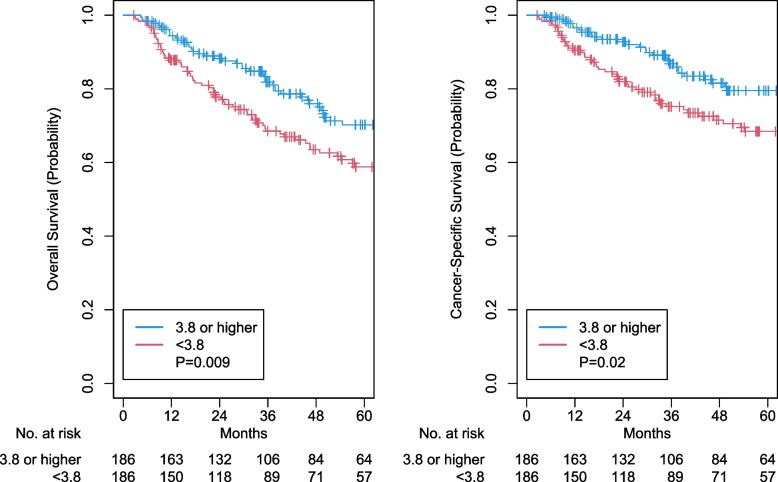
Kaplan–Meier curves for overall and cancer-specific survival outcomes for low versus high lymphocyte-monocyte ratio. LMR: lymphocyte-monocyte ratio

**Fig. 3:**
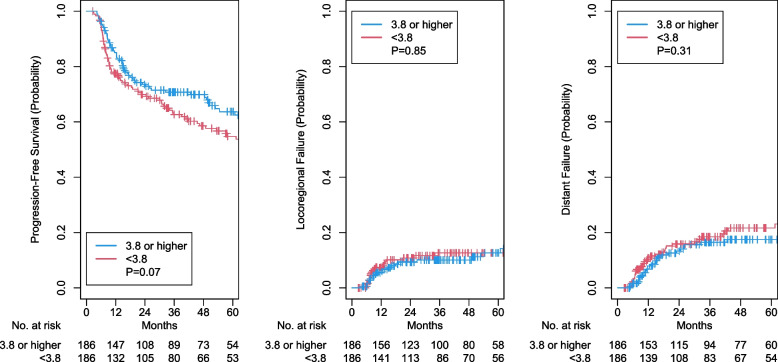
Kaplan–Meier curves for progression-free survival and cumulative incidence of locoregional and distant failure outcomes for low versus high lymphocyte-monocyte ratio. LMR: lymphocyte-monocyte ratio

After propensity score matching, a total of 186 pairs were matched, and their baseline characteristics were well balanced (Table 1). Lower LMR remained to be associated with worse OS (HR 1.59, 95% CI 1.12–2.26, *p* = 0.009; Fig. 2) and CSS (HR 1.68, 95% CI 1.08–2.63, *p* = 0.02; Fig. 2). However, it was not associated with PFS (aHR 1.35, 95% CI 0.97–1.86, *p* = 0.07), LRF (aHR 1.06, 95% CI 0.58–1.94, *p* = 0.85), or DF (aHR 1.30, 95% CI 0.78–2.17, *p* = 0.31; Fig. 3).

For the entire cohort, median ANC was 4750 cells/microliter (interquartile range 3607–6282). When the absolute neutrophil count as a continuous variable was adjusted in the MVA, similar findings for the LMR were noted on MVA. Higher LMR was associated with improved OS (aHR 0.91, 95% CI 0.83–1.00, *p* = 0.047) and CSS (aHR 0.85, 95% CI 0.74–0.98, *p* = 0.02), while it was not associated with PFS (aHR 0.95, 95% CI 0.88–1.02, *p* = 0.17), LRF (aHR 0.91, 95% CI 0.77–1.07, *p* = 0.25), or DF (aHR 0.95, 95% CI 0.83–1.09, *p* = 0.48). In the subgroup of 319 patients (67.0%) with available HPV data for oropharyngeal cancer, 231 patients (48.5%) had HPV-associated head and neck cancer. LMR status was not associated with both OS and CSS regardless of HPV status (Table 5).

**Table 5:** Cox multivariable analysis for overall survival and cancer-specific survival stratified by p16 status

p16-negative cohort						
	Overall survival			Cancer-specific survival		
	aHR	95% CI	*P*	aHR	95% CI	*P*
LMR						
3.8 or higher	Reference			Reference		
< 3.8	0.87	0.42–1.83	0.72	1.28	0.50–3.31	0.61
p16-positive cohort						
	Overall survival			Cancer-specific survival		
	aHR	95% CI	*P*	aHR	95% CI	*P*
LMR						
3.8 or higher	Reference			Reference		
< 3.8	1.26	0.71–2.26	0.43	1.49	0.69–3.22	0.31

*LMR* lymphocyte-monocyte ratio, *aHR* adjusted hazards ratio, *95% CI* 95% confidence interval

## Discussion

To our knowledge, this is the first study of a North American head and neck cancer patient cohort to evaluate the prognostic value of LMR. Low LMR, both as a continuous variable and dichotomized variable below the median value, was associated with worse OS and CSS. Low LMR was associated with higher T staging and negative HPV status.

The association of LMR with survival outcomes and higher T staging in our study is inconsistent with a recent meta-analysis evaluating the role of LMR as a prognostic factor among patients with head and neck cancer [4]. Many studies included in the meta-analysis were performed outside the North America, and a recent Korean study showed different average LMR across age and sex groups in healthy subjects, suggesting varied degrees of the prognostic role for LMR based on different patient demographics [18].

Our finding on low LMR as an adverse prognostic factor supports a growing body of literature that systemic inflammation, as indicated by inflammatory markers, has been demonstrated to result in worse prognosis [19]. Recent studies have emphasized that host inflammatory response greatly influences the development of cancer, as it has been suggested that inflammatory cells and cytokines are increasingly likely to impact cancer growth and metastasis, while contributing to immunosuppression associated with malignancy [20, 21]. Peripheral blood biomarkers have been used to capture the magnitude of such inflammation, and several studies have demonstrated their prognostic value across cancer types [22]. An insufficient count of lymphocytes can result in inadequate immunological response to a tumour present, promoting progression and spread; specifically, it has been reported that types of tumor infiltrating lymphocytes, including CD8 + T cells and memory T cells, are associated with positive prognosis of tumors [23]. Increased monocyte number, however, has been associated with unfavorable outcomes of a variety of tumors, differentiating into tumor-associated macrophages and promoting tumor angiogenesis, growth, invasion, and migration [23]. Our cutoff of 3.8 as a median value in this study is consistent with previous studies incorporating cutoff values ranging from 2.35 to 5.22 [24].

Low LMR was also associated with HPV-negative cancer. HPV positive cancers have a distinct molecular pathogenesis from HPV negative cancers facilitated by upregulation of p16 [8, 25]. One study found increased CD8 + T cell tumor infiltration in HPV positive cancer compared to HPV negative tumors [26]. The different tumor microenvironments between the head and neck cancer subgroups may in part explain our findings. Another study found that HPV can inhibit monocyte differentiation to Langerhans cells, thereby evading immune surveillance [27]. It is possible that through this mechanism, a higher proportion of monocytes would be insignificant in affecting outcome.

### Limitations

The limitations of this study are those inherent to single-institution retrospective studies including potential for selection bias. In addition, our analysis did not include address change in pre-treatment compared to post-treatment LMR (delta LMR), which may better account for baseline LMR and be a stronger predictor of prognosis [28]. Since only those with definitive chemoradiation were included in this study, our findings may not be generalizable to other patient populations treated with surgery, postoperative radiation, surgery or radiation alone, and palliative radiation.

## Conclusion

Low LMR, both as a continuous variable and dichotomized variable below 3.8 in our study, was associated with worse overall survival and cancer-specific survival. Low LMR was associated with higher T staging and HPV negative cancer. Further studies are warranted to elucidate the role of inflammatory markers in head and neck cancer management.

## Data Availability

Data cannot be shared publicly because of protected health information. Data are available from the Institutional Data Access / Ethics Committee for researchers who meet the criteria for access to confidential data. Research data will be shared upon request to the corresponding author.
